# Reducing the Effect of Positioning Errors on Kinematic Raw Doppler (RD) Velocity Estimation Using BDS-2 Precise Point Positioning

**DOI:** 10.3390/s19133029

**Published:** 2019-07-09

**Authors:** Shunli Duan, Wei Sun, Chenhao Ouyang, Xinyu Chen, Junbo Shi

**Affiliations:** 1 School of Geomatics, Liaoning Technical University, Fuxin 123000, China; 2 School of Geodesy and Geomatics, Wuhan University, Wuhan 430079, China

**Keywords:** BDS-2, PPP, raw Doppler, velocity estimation

## Abstract

In the traditional raw Doppler (RD) velocity estimation method, the positioning error of the pseudorange-based global navigation satellite system (GNSS) single point positioning (SPP) solution affects the accuracy of the velocity estimation through the station-satellite unit cosine vector. To eliminate the effect of positioning errors, this paper proposes a carrier-phase-based second generation of the BeiDou navigation satellite system (BDS-2) precise point positioning (PPP) RD velocity estimation method. Compared with the SPP positioning accuracy of tens of meters, the BDS-2 kinematic PPP positioning accuracy is significantly improved to the dm level. In order to verify the reliability and applicability of the developed method, three dedicated tests, the vehicle-borne, ship-borne and air-borne platforms, were conducted. In the vehicle-borne experiment, the GNSS and inertial navigation system (INS)-integrated velocity solution was chosen as the reference. The velocity accuracy of the BDS-2 PPP RD method was better than that of SPP RD by 28.4%, 27.1% and 26.1% in the east, north and up directions, respectively. In the ship-borne and air-borne experiments, the BDS-2 PPP RD velocity accuracy was improved by 17.4%, 21.4%, 17.8%, and 38.1%, 17.6%, 17.5% in the same three directions, respectively, compared with the BDS-2 SPP RD solutions. The reference in these two tests is the real-time kinematic (RTK) Position Derivation (PD)-based velocity.

## 1. Introduction

The position derivation (PD), raw Doppler (RD), carrier-phase-derived Doppler (DD) and time-differenced carrier phase (TDCP) methods are four major velocity estimation approaches for satellite-based applications. The PD velocity estimation method makes use of the first-order derivation of consecutive positions; the RD velocity estimation method refers to the raw Doppler measurements for velocity determination; the DD method utilizes the Doppler measurements derived from carrier phase observations, rather than the Raw Doppler measurement in the RD method, at two consecutive epochs to calculate the velocity; and the TDCP method takes advantage of the pseudorange and carrier phase observations at two consecutive epochs to determine the velocity.

Previous studies prove that the velocity estimation accuracy of the PD method is highly vulnerable to the positioning accuracy and the moving condition. For example, He et al. [1] analyzed the differential GPS (DGPS) PD velocity estimation method by airborne experiments, and the maximum velocity error reached 2 m/s in the airplane taking-off stage. Li et al. [2] performed a comprehensive velocity estimation analysis based on three global navigation satellite system (GNSS) positioning modes, including single point positioning (SPP), pseudorange-based DGPS and carrier-phase-based real-time kinematic (RTK) in Antarctica. Experimental results showed that the RTK velocity solution was the best, with an accuracy of 3 mm/s in the vertical direction. Meanwhile, the velocity accuracy of the combined GPS/BeiDou navigation satellite system (BDS) solution was improved by 39% over that of the GPS-only solution.

Both the RD and DD methods are Doppler observation-based velocity estimation methods [3]. Their difference is that the RD method uses the raw Doppler observation, whereas the carrier-derived Doppler observation is adopted in the DD method. A series of studies have shown that the RD method is more suitable for kinematic applications and the DD method is more suitable for static or low kinematic applications (e.g., seismic monitoring). Wang et al. [3] compared the GPS RD and DD methods. Their Root Mean Square (RMS) statistics in the GPS static experiment were 1.1, 1.3, 2.7 cm/s and 1.5, 1.5, 2.7 mm/s, respectively, in the east (E), north (N), up (U) directions. In the airborne experiment, the RMS statistics of the RD and DD methods with respect to the inertial navigation system (INS) velocity solution were 13.9, 9.9, 6.8 cm/s and 14.4, 10.7, 8.1 cm/s, respectively, for the three directions. Liu et al. [5] compared the BDS SPP RD and DD velocity estimation methods. Their RMS values in the static experiment were 1.5, 2.0, 4.7 cm/s and 1.8, 2.6, 6.3 mm/s for the RD and DD method, respectively. In the vehicle-borne experiment, the BDS SPP RD method’s RMSs were 4.4, 5.8, 5.1 cm/s with respect to the INS reference, whereas the BDS SPP DD method was 7.2, 7.8, 10.1 cm/s. Zheng et al. [6] analyzed the BDS-only and GPS-only SPP RD velocity estimation method, whose stability and accuracy could be remarkably improved by the combination of GPS/BDS. Li et al. [7] applied the RD method to GPS kinematic positioning, which improved the ambiguity fixed rate and positioning accuracy. Zhang [8] and Li [9] adopted the GPS DD method for seismic monitoring. Results showed that the velocity estimated by the DD method was in good agreement with the strong motion seismograph record. In addition, Zhang [10] investigated the influence of GPS receiver clock jumps on DD velocity, with an accuracy of 0.2, 0.1, 0.5 m/s in the E, N, U directions, respectively. Ye et al. [11] analyzed the BDS SPP-based velocity with the RD and DD methods. The static data showed that the RD accuracy was related to the receiver type, while the RMS value of the DD method attained 2, 5, 8 mm/s in the E, N, U directions, respectively. In the vehicle-borne experiment compared with the INS, the E, N, U RMS statistics of RD and DD methods achieved 3.7, 4.5, 2.8 cm/s and 4.2, 4.7, 2.8 cm/s, respectively.

The TDCP method was first proposed by Graas [12] in 2004. Wendel et al. [13] and Soon et al. [14] applied this method to GPS/INS integrated navigation, which improved the velocity estimation accuracy in the integrated system. Freda et al. [15] investigated the jump of velocity errors by eliminating the effect of the satellite position switch between two consecutive broadcast ephemeris. The GPS static experiment proved that the velocity accuracy of this method could reach the order of mm/s. Ye et al. [11] analyzed the BDS velocity estimation with the TDCP method, and the RMSs of static velocity errors were 2, 4, 7 mm/s in the E, N, U directions, respectively. Compared with the INS solution, the external E, N, U RMSs in the kinematic experiment were 2.4, 2.9, 2.6 cm/s, respectively.

Table 1 summarizes the abovementioned four satellite-based velocity estimation methods. From Table 1, it can be seen that due to the fact of the average velocity between two consecutive epochs, the PD, DD and TDCP methods may cause large errors under complex kinematic conditions such as starting, braking and turning movements. Instead, only the RD method estimates the instantaneous velocity of the moving object based on Doppler observations at the instantaneous epoch, thus is more suitable for real-time navigation applications with complex motions.

It should be noted that most existing researches on the RD velocity estimation method are based on the SPP solution, which could reach 20 m or more positioning error under kinematic conditions. This large positioning error reduces the RD velocity accuracy through the line-of-sight vector between the user receiver on the ground and the satellite in the space. On the other hand, RTK can obtain cm-level kinematic positioning accuracy [16,17], but the RTK technology requires at least two receivers with a higher economical cost. In addition, the RTK positioning accuracy decreases as the distance increases, which then prevents the long-distance navigation application. Compared with the SPP and RTK modes, the precise point positioning (PPP) technology can achieve centimeter-level kinematic positioning accuracy by utilizing a single receiver and high-precision orbit/clock products without any restriction of a base receiver and the distance [18,19,20,21,22].

In order to reduce the SPP positioning error influence on the RD velocity estimation and also reduce the economical cost of the RTK mode, a kinematic velocity estimation method based on BeiDou Navigation Satellite System (BDS-2) PPP is proposed in this paper. Compared with the SPP technology, the proposed PPP accuracy is significantly better. Meanwhile, economical costs of the RTK technology can be eliminated. In the following sections, the proposed BDS-2 PPP RD velocity estimation method is first explained. Then, experiments in three scenarios (vehicle-born, ship-born and air-borne) are described and experimental results are discussed. Finally, some conclusions drawn from this work are given in the last section.

## 2. Methodology of the Proposed BDS-2 PPP-Based RD Velocity Estimation

**Figure 1:**
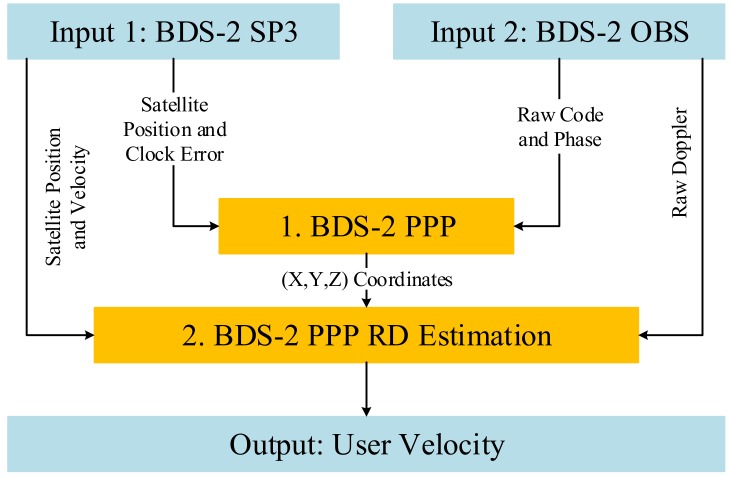
Flowchart of the proposed BeiDou Navigation Satellite System (BDS-2) precise point positioning (PPP) raw Doppler (RD) velocity estimation algorithm.

Figure 1 shows the implementation flowchart of the proposed BDS-2 kinematic PPP RD method. The algorithm, requiring BDS-2 precise ephemeris and pseudorange/phase/Doppler observations as inputs, includes the following two steps:

Step 1. User coordinates are obtained using BDS-2 pseudorange/phase observations and precise ephemeris. The BDS-2 PPP function model can be expressed as [23]:

(1) $$\left\{\begin{array}{l} P=\rho +c\left(dt_{r}-dt^{s}\right)+dtrop+dion+b_{P}^{r}-b_{P}^{s}+\varepsilon_{P}\\ L=\rho +c\left(dt_{r}-dt^{s}\right)+dtrop-dion+\lambda N+b_{L}^{r}-b_{L}^{s}+\varepsilon_{L} \end{array}\right.$$

where P is the raw pseudorange measurement, L is the raw carrier phase measurement, ρ is the geometric distance between receiver and satellite, c is the speed of light in vacuum, $dt_{r}$ is the receiver clock error, $dt^{s}$ is the BDS satellite clock error, dion and dtrop represent ionospheric delay and tropospheric delay, respectively, λ is wavelength, N is the integer ambiguity, $b_{p}^{r}$ is the receiver code hardware delay (bias), $b_{p}^{s}$ is the satellite code hardware delay (bias), $b_{L}^{r}$ is the receiver carrier phase hardware delay (bias), $b_{L}^{s}$ is the satellite carrier phase hardware delay (bias), $\varepsilon_{P}$ is pseudorange measurement noise and $\varepsilon_{L}$ is carrier phase measurement noise. It should be noted that the ionospheric error is normally eliminated by an ionosphere-free observation combination based on Equation (1).

Step 2. Send the BDS-2 PPP coordinates to the RD velocity estimator to calculate the user velocity. The equation of the BDS-2 RD method is expressed as:

(2) $$\lambda D_{r}^{s}=\dot{\rho }+c\left(\dot{d}t_{r}-d{\dot{t}}^{s}\right)+\dot{d}trop-\dot{d}ion+\varepsilon_{D}$$

where:

(3) $$\dot{\rho }=e_{r}^{s}\cdot \left({\dot{r}}^{s}-{\dot{r}}_{r}\right)$$

(4) $$e_{r}^{s}=\frac{r^{s}-r_{r}}{\rho }$$

where $D_{r}^{s}$ is raw Doppler measurement, $\dot{\rho }$ is the range-rate of ρ, $\dot{d}t_{r}$ is the receiver clock drift, $\dot{d}t^{s}$ is the BDS satellite clock drift, $\dot{d}trop$ and $\dot{d}ion$ represent the rates of tropospheric delay and ionospheric delay, respectively, $r^{s}$ and ${\dot{r}}^{s}$ are satellite position and velocity vectors calculated from precise ephemeris, respectively, $r_{r}{=[X,Y,Z]}^{T}$ is the receiver position vector obtained in Step 1, ${\dot{r}}_{r}$ is the receiver velocity vector, $e_{r}^{s}$ is the line-of-sight unit vector between receiver and satellite and $\varepsilon_{D}$ is Doppler measurement noise. As the interval for kinematic velocity estimation is generally small, the atmospheric delay rates $\dot{d}trop$ and $\dot{d}ion$ in Equation (2) can be neglected.

Denote the number of visible satellites as n. The least-squares estimation is utilized for the calculation of three-dimensional velocity ${\dot{r}}_{r}=[V_{x},V_{y},V_{z}{]}^{T}$ in Equation (3) and the receiver clock drift $\dot{d}t_{r}$ in Equation (2):

(5) $$X={\left(B^{T}PB\right)}^{-1}B^{T}PL$$

where: $X_{4*1}=\left[\begin{array}{c} V_{x}\\ V_{y}\\ V_{z}\\ c\cdot \dot{d}t_{r} \end{array}\right],B_{n*4}=\left[\begin{array}{cccc} -e_{x}^{1} & -e_{y}^{1} & -e_{z}^{1} & 1\\ -e_{x}^{2} & -e_{y}^{2} & -e_{z}^{2} & 1\\ \vdots & \vdots & \vdots & \vdots \\ -e_{x}^{n} & -e_{y}^{n} & -e_{z}^{n} & 1 \end{array}\right],L_{n*1}=\left[\begin{array}{c} \lambda \cdot D_{r}^{1}-e_{r}^{1}\cdot {\dot{r}}^{1}+c\cdot \dot{d}t^{1}\\ \lambda \cdot D_{r}^{2}-e_{r}^{2}\cdot {\dot{r}}^{2}+c\cdot \dot{d}t^{2}\\ \vdots \\ \lambda \cdot D_{r}^{n}-e_{r}^{n}\cdot {\dot{r}}^{n}+c\cdot \dot{d}t^{n} \end{array}\right]$ and $P_{n*n}=\left[\begin{array}{ccc} \sin^{2}E & & \\ & \ddots & \\ & & \sin^{2}E \end{array}\right]$ with E as the satellite elevation angle.

Through Equations (3)–(5), we can see that the positioning error will contaminate matrices L and B by affecting the station-satellite unit cosine vector, which subsequently increases the velocity estimation error. This is the motivation of our study to utilize the high-precision BDS-2 PPP algorithm to reduce the positioning error impact on the velocity estimation in the traditional SPP approach.

## 3. Experiments and Analysis

In order to verify the reliability of the proposed method and its applicability in various kinematic platforms, three kinematic experiments on the vehicle-borne, ship-borne and air-borne platforms are carried out in this paper. Table 2 lists some details of the three experiments. The vehicle-borne experiment used a NovAtel OEM4 receiver with a sampling rate of 1 Hz for 10 min and the acquisition location was in Fuxin, Liaoning, China. The ship-borne experiment was carried out in Wuhan, East Lake using a ComNav K505 receiver with a sampling frequency of 1 Hz. The air-borne experiment used a NovAtel OEM4 receiver with a sampling frequency of 5 Hz for 62 min and the acquisition location was in Dengfeng, Henan, China. Reference receivers were set up in all three experiments. Among the three, the vehicle-borne experiment utilized BDS/GPS RTK + INS integrated velocity as the reference [24], whereas both the ship-borne and the air-borne experiments used the velocity calculated by the BDS/GPS RTK PD method as the reference. 

### 3.1. Vehicle-Borne Experiment

**Figure 2:**
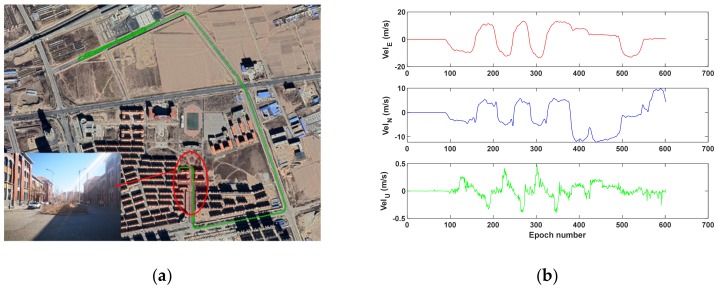
Vehicle’s trajectory (**a**) and reference velocity (**b**); the red circle marks the observation condition with surrounded buildings and trees.

The average number of observed BDS-2 satellites is 7.6 (~3.7 GEO, ~3.9 IGSO, 0 MEO), and the average position dilution of precision (PDOP) is 4.2 during 10 min. It should be mentioned that all tests in this study only utilize 14 BDS-2 satellites, rather than MEO satellites contained in BDS-3 [25]. Figure 2 shows the vehicle’s trajectory (a) and the reference velocity (b). It can be seen from Figure 2b that the velocity in the east, north and up directions are within 20, 10 and 0.5 m/s, respectively.

**Figure 3:**
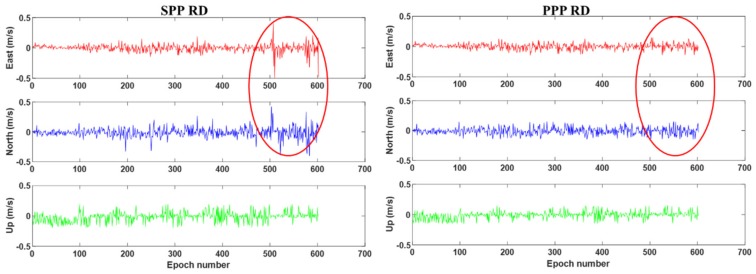
BDS-2 single point positioning (SPP) (**left**) and PPP (**right**) RD velocity errors of the vehicle–borne experiment. The red circle marks epochs blocked by buildings and trees.

Figure 3 displays the velocity error computed by BDS-2 SPP RD (left) and BDS-2 PPP RD (right) results. It can be seen from Figure 3 that the accuracy of the BDS PPP RD method in the vehicle-borne experiment is significantly better than that of the SPP RD method. It is noted that during the 500th–600th epoch, when satellite signals are partially blocked by surrounding buildings and trees, the velocity error of BDS-2 SPP RD fluctuates largely with a maximum error of 0.5 m/s. In contrast, the BDS-2 PPP RD velocity is obviously less affected by the observation environment compared to the SPP RD solution, which proves that the method proposed in this paper is more suitable in complex kinematic scenes. The RMS statistics of the SPP RD and PPP RD velocities are summarized in Table 3. It can be seen from Table 3 that the precision of both the PPP RD method and the SPP RD methods can reach the level of cm/s. The velocity accuracies of the the PPP RD method are 28.4%, 27.1%, 26.1% better than the SPP RD method in the E, N, U directions, respectively.

### 3.2. Ship-Borne Experiment

**Figure 4:**
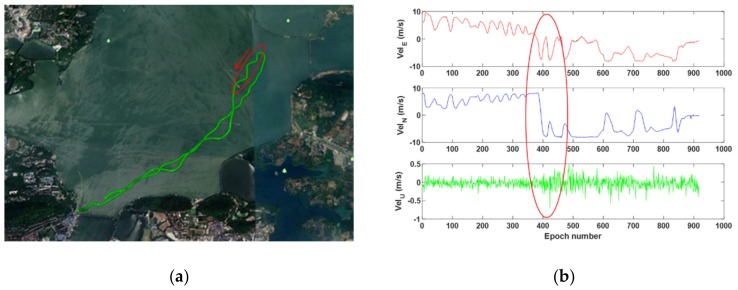
Ship’s trajectory (**a**) and reference velocity (**b**).

The trajectory (a) of the ship-borne experiment and its reference velocity (b) are shown in Figure 4. The average number of observed satellites is 9 (GEO = 5, IGSO = 3, MEO = 1), and the average PDOP is 2.2 during 16 min. Since the ship used in this experiment is a motorboat, the magnitude and direction of its velocity can change must faster than that in the vehicle-borne test. The motorboat takes a U-turn and several consecutive sharp turns between the 370th and 400th epochs, which are marked in red in Figure 4.

**Figure 5:**
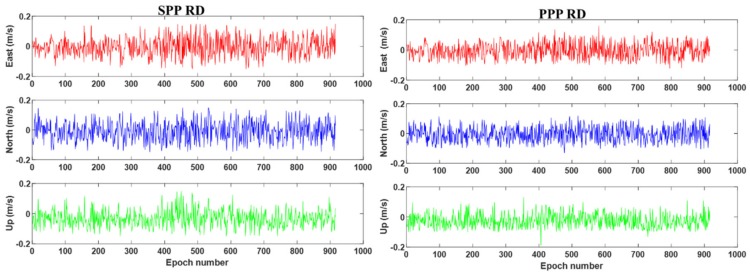
BDS-2 SPP (**left**) and PPP (**right**) RD velocity errors of ship–borne experiment.

Figure 5 presents the velocity errors calculated by the BDS-2 SPP and PPP RD methods. It can be seen from Figure 5 that the accuracy of the BDS PPP RD method in the ship-borne experiment is much better than that of the SPP RD method, especially between the 370th and 400th epochs when several turns occur. The RMS statistics of SPP and PPP RD are shown in Table 4. We can see from Table 4 that the precision of the PPP and SPP RD methods can reach the cm/s level in the ship-borne experiment. Moreover, the velocity estimated by the PPP RD method is better than that of the SPP RD method by about 1.0 cm/s, and the improvement rates are 17.4%, 21.4%, 17.8% in the E, N, U directions, respectively.

### 3.3. Air-Borne Experiment

**Figure 6:**
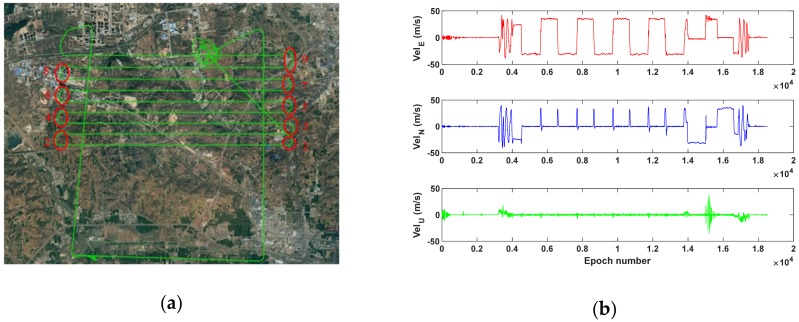
Airplane’s trajectory (**a**) and reference velocity (**b**). Red circles are markers for nine airplane turns.

The average number of observed satellites is 12.2 (~4.8 GEO, ~4.6 IGSO, ~2.8 MEO), and the average PDOP is 2.4 during 62 min. Figure 6a displays the trajectory. In this experiment, we use fixed-wing unmanned aviation vehicle (UAV) which experiences four stages: Static, taking-off, cruising and landing. The switch among these four stages could be clearly seen from the velocity change in Figure 6b. In addition, the aircraft’s velocity is approximately twice the velocity of the land vehicle and the ship, with a maximum close to 50 m/s.

**Figure 7:**
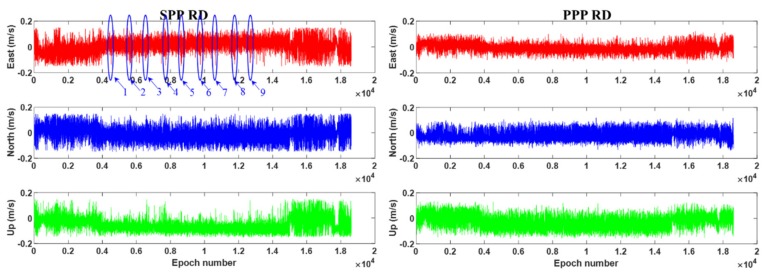
BDS-2 SPP (**left**) and PPP (**right**) RD velocity errors of air-borne experiment. Blue circles in the left plot denote nine airplane turns.

**Figure 8:**
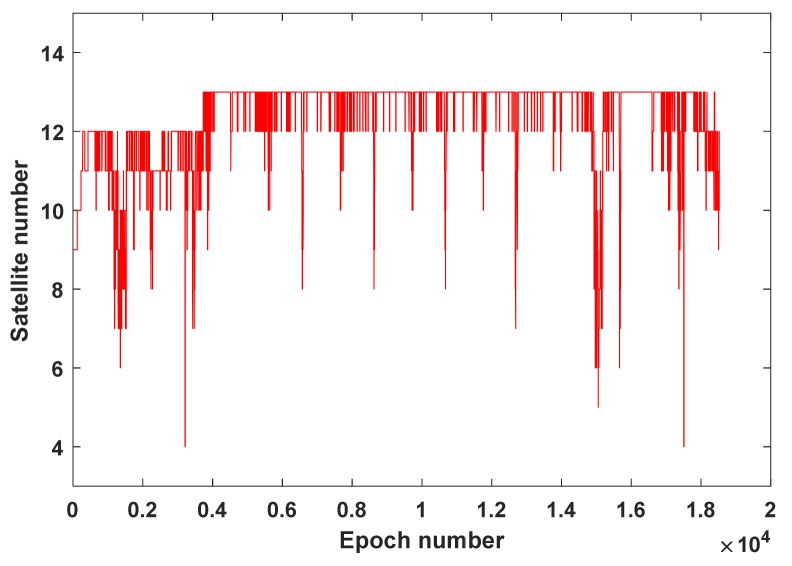
Number of observed satellites of air–borne experiment.

Figure 7 shows the error series obtained by the BDS-2 SPP and PPP RD methods, separately. It can be concluded from Figure 7 that the PPP RD method is obviously better than the SPP RD method, especially during the taking-off and landing stages. Moreover, during the nine turns in the cruising stage, on the one hand, the number of observed satellites suddenly decreases due to the sudden motion change of the airplane (Figure 8); on the other hand, owing to the sudden change in the velocity magnitude and direction, the SPP RD method has a large error in the velocity solution, especially in the east–west direction as highlighted in Figure 6. The RMS statistics of SPP RD and PPP RD are shown in Table 5. It is clear from Table 5 that the precision of the PPP RD method is better than that of the SPP RD method, and the improvement rates are 38.1%, 17.6%, 17.5% in the E, N, U directions, respectively. 

**Figure 9:**
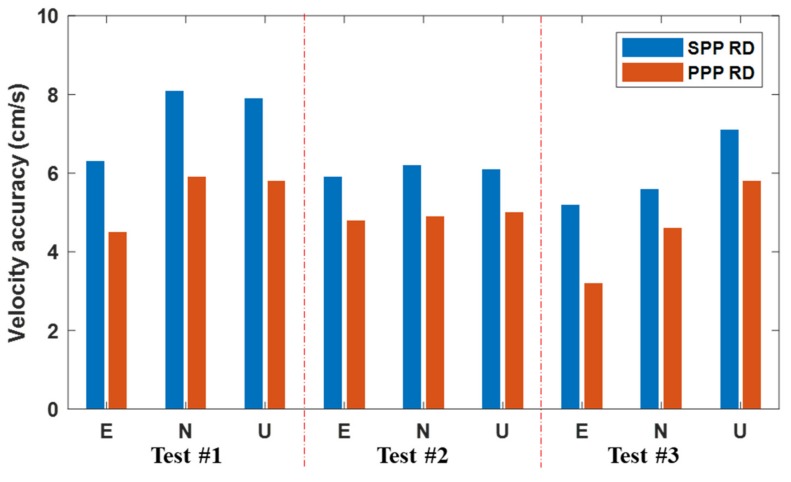
Velocity accruracy comparison between the PPP and SPP mode.

From the above three experiments, it can be concluded from Figure 9 that the BDS-2 PPP RD method has better velocity estimation precision than the SPP RD method in various kinematic scenarios by 1–2 cm/s in the three directions. The vehicle-borne experimental result shows that when the observation environment becomes worse, the PPP RD method can significantly improve the velocity estimation performance. Through ship-borne and air-borne experiments, it can be seen that, when the magnitude and direction of the moving object’s velocity suffer from active changes, the PPP RD method can also provide more precise velocity than the SPP RD method.

## 4. Conclusions

Among the four velocity estimation methods, the RD method is more suitable for kinematic velocity estimation applications with complex movements. Most existing RD velocity estimation research is based on the SPP, whose large positioning errors subsequently affect the accuracy of the velocity estimation. In order to reduce the influence of the positioning error on RD velocity results, this paper proposes an RD kinematic velocity estimation method based on BDS-2 PPP, and gives the specific implementation procedure.

The BDS-2 PPP RD method proposed in this paper was verified by experiments in three different scenarios: Vehicle-borne, ship-borne and air-borne. The results show that: (1) The BDS-2 PPP RD method is not affected by the complex movements including starting, braking and turning; (2) in three kinematic experiments, the velocity precision of the BDS-2 PPP RD method is 1–2 cm/s and the improvement rates are 17%–38% in the E, N, U directions compared with the BDS-2 SPP RD method. It is worth mentioning that the BDS-2 PPP algorithm is sophisticated and easy to implement. As a result, the application of this technology in kinematic velocity estimation applications does not induce any hardware or software cost, and thus is worth popularization.

## Figures and Tables

**Table 1:** Comparison of four velocity estimation methods.

Method	Static (RMS)	Kinematic (RMS)	Estimated Velocity
PD	mm/s–cm/s	cm/s–m/s	Average between two epochs
RD	mm/s–cm/s	mm/s–cm/s	Instantaneous at current epoch
DD	mm/s	cm/s–dm/s	Average between two epochs
TDCP	mm/s	cm/s–dm/s	Average between two epochs

**Table 2:** Experimental description.

	Vehicle-Borne	Ship-Borne	Air-Borne
UTC	2018/11/6/ 7:04–7:14	2016/6/14/ 8:16–8:32	2016/6/15/ 8:00–9:02
Location	42°02′ N 121°39′E	30°32′N 114°23′E	34°25′N 113°05′E
Sampling	1 Hz	1 Hz	5 Hz
Receiver	NovAtel OEM4	ComNav K505	NovAtel OEM4
Velocity	~20 m/s	~15 m/s	~40 m/s
Reference	BDS/GPS RTK + INS	BDS/GPS RTK PD	BDS/GPS RTK PD

**Table 3:** RMS statistics and comparison of the vehicle–borne experiment.

	SPP RD (cm/s)	PPP RD (cm/s)	Improvement (%)
East	6.3	4.5	28.4
North	8.1	5.9	27.1
Up	7.9	5.8	26.1

**Table 4:** RMS statistics and comparison of the ship–borne experiment.

	SPP RD (cm/s)	PPP RD (cm/s)	Improvement (%)
East	5.9	4.8	17.4
North	6.2	4.9	21.4
Up	6.1	5	17.8

**Table 5:** RMS statistics and comparison of air–borne experiment.

	SPP RD (cm/s)	PPP RD (cm/s)	Improvement (%)
East	5.2	3.2	38.1
North	5.6	4.6	17.6
Up	7.1	5.8	17.5
